# ODEbase: a repository of ODE systems for systems biology

**DOI:** 10.1093/bioadv/vbac027

**Published:** 2022-04-26

**Authors:** Christoph Lüders, Thomas Sturm, Ovidiu Radulescu

**Affiliations:** 1 Institute of Mathematics, University of Kassel, 34132 Kassel, Germany; 2 CNRS, Inria and the University of Lorraine, 54506 Nancy, France; 3 MPI Informatics and Saarland University, 66123 Saarbrücken, Germany; 4 Laboratory of Pathogen Host Interactions UMR 5235, University of Montpellier and CNRS, 34095 Montpellier, France

## Abstract

**Summary:**

Recently, symbolic computation and computer algebra systems have been successfully applied in systems biology, especially in chemical reaction network theory. One advantage of symbolic computation is its potential for qualitative answers to biological questions. Qualitative methods analyze dynamical input systems as formal objects, in contrast to investigating only part of the state space, as is the case with numerical simulation. However, corresponding tools and libraries have a different set of requirements for their input data than their numerical counterparts. A common format used in mathematical modeling of biological processes is Systems Biology Markup Language (SBML). We illustrate that the use of SBML data in symbolic computation requires significant pre-processing, incorporating external biological and mathematical expertise. ODEbase provides suitable input data derived from established existing biomodels, covering in particular the BioModels database.

**Availability and implementation:**

ODEbase is available free of charge at https://odebase.org.

## 1 Introduction

Symbolic computation is a well-established field in mathematics and computer science that has a strong community and numerous applications in science. In contrast to numerical computation, which uses approximate floating point numbers, symbolic computation works with exact mathematical expressions.

Recently, symbolic computation methods are playing an increasing role in systems biology and mathematical biology. Problems investigated using such methods include sustained oscillations and Hopf bifurcations, multi-stationarity, multi-scale model reduction, dynamical invariants and structural properties of steady state varieties such as, for example, binomiality and toricity; for details see Boulier *et al.* (2018), and the references there. Compared to numerical analysis and simulation, symbolic computation provides not only quantitative but also qualitative results about network dynamics, to some extent in parametric settings. The biological systems investigated so far had a focus on reaction networks in the sense of chemical reaction network theory (Aris, 1965; Feinberg, 2019). Such networks are usually stored and exchanged in the Systems Biology Markup Language (SBML), a free, open and standardized XML-based format (Hucka *et al.*, 2003).

On the one hand, symbolic computation does not utilize the full information contained in SBML models. For instance, SBML was designed with a focus on network simulation and supports corresponding concepts like events and initial assignments, which are not natural from a formal symbolic computation point of view. On the other hand, symbolic computation operates on formal objects like polynomials, rational numbers, matrices and ordinary differential equations (ODEs), which are not readily available in SBML. For instance, ODEs, describing differential network kinetics along with algebraic constraints, such as conservation laws, can be considered either as pieces of code to be used with numerical solvers or as mathematical expressions to be studied with symbolic computation. The genuine difference between dynamic simulation and static formal analysis requires sensitivity to details and rigor in the course of the construction of symbolic computation input from available SBML descriptions. It is noteworthy that existing SBML parsers generate input for numerical simulation, which is not suited for symbolic computation. MathSBML (Shapiro *et al.*, 2004), SBFC (Rodriguez *et al.*, 2016), SBMLtoODEpy (Ruggiero and Versypt, 2019) and SBML2Modelica (Maggioli *et al.*, 2020) fall into that category.

## 2 Approach

It is important to understand that the rigorous construction of symbolic computation input poses some substantial problems. Solving, or even recognizing, such problems, requires joint competence and combined efforts not only from biology but also including mathematics and computer science. We give some examples:

- SBML allows floating-point values for various entities. However, floating-point values exhibit representation errors and computations are prone to rounding errors. This is inadequate for symbolic computation, where exact computations are performed.
- SBML has liberal naming conventions for species and parameters that interfere with the typically strict rules of symbolic computation software, which are oriented toward mathematical notation. If different users of symbolic computation software rename those identifiers at their own discretion, it becomes cumbersome to compare their results.
- SBML gives modelers versatile opportunities of expression, such as local parameters, function definitions, rules and initial assignments. For practical reasons, scientific software does not generally support the full SBML feature set. This leads to incompletely imported models or it prohibits the import entirely.
- Symbolic computation is concerned with mathematical properties like deficiency and linear conservation laws, which are available in SBML only implicitly through computation. Explicit availability is desirable, especially, since some of those computations can become surprisingly time-consuming.

Although integration of symbolic computation input into SBML might appear natural, there are obstacles on both sides. On the symbolic computation side, established software is usually general-purpose, and systems biology is not yet a strong focus of the community. Therefore, widespread support of SBML as an input format for symbolic computation software cannot be expected in the near future. On the systems biology side, the SBML standard would need to be extended. Standardization generally requires considerable efforts, and it seems unlikely that this will be pursued before the links between symbolic computation and systems biology have been further strengthened.

The interdisciplinary project SYMBIONT brings together researchers from mathematics, computer science, and systems biology (Boulier *et al.*, 2018). Within SYMBIONT, we have started an online database *ODEbase*, which collects symbolic computation input for existing SBML models. All our models originate from the BioModels database (Le Novère *et al.*, 2006). Out of the 1044 models from the curated branch of BioModels, we have currently compiled 662 into ODEbase. As ODEbase has turned out to be extremely valuable throughout the SYMBIONT project, we now make it available beyond the lifespan of the project to the computational algebra and systems biology communities as a free and open database at https://odebase.org. By doing so, we expect to promote systems biology models to researchers developing general purpose symbolic computation methods and also facilitate the benchmarking of such methods. If models require updates, revised versions will be made available, keeping all previous versions for reference. Data can be extracted in Maple, Reduce, SageMath and LATEX format. We are open to supporting further formats in the future.

## 3 The content of ODEbase data sets

For each model in ODEbase, the following dataset is computed from the original SBML input. The ODEs and the constraints mentioned below together establish the *ODE systems* referred to in the title.

- *Stoichiometric and kinetic matrices*: Stoichiometric and kinetic matrices are made explicit using exact rational numbers.
- *ODEs for species concentrations*: These are explicit first-order, non-linear ODEs that are often, but not necessarily, autonomous. Species are named *x*_1_, …, *x_n_*, following common mathematical notation. If species rate rules are present in the model, the corresponding ODEs are included as well.
- *Parameter values*: Our naming of parameters follows common mathematical notation, viz., *k*_1_, …, *k_m_*. Assignment rules, initial assignments and initial concentrations are taken into consideration.
- *Map between ODEbase names and original model names*: A bijective mapping between the mathematical names for species and parameters and their respective SBML names is provided.
- *Constraints*: All SBML species assignment rules are converted to formal constraints. Furthermore, linear conservation constraints are computed using an extension of Schuster and Höfer (1991).
- *Deficiency*: The deficiency is computed from the network’s complexes. This is a measure of how independent the reaction vectors are, given the network’s linkage class structure (Feinberg, 2019, Sect. 6.3).
- *Classification*: Polynomials and, more generally, rational functions play a crucial role in symbolic computation. We classify whether ODE vector fields and constraints are covered by such expressions. In the polynomial case, we furthermore check if the SBML-specified kinetics differs from the regular mass-action kinetics (Feinberg, 2019, Sect. 2.1.2) only by a constant factor. This is a conservative heuristic for identifying models with mass-action kinetics.

All models in ODEbase are a faithful conversion from the respective SBML model. SBML features recognized during the conservation process include species with boundary condition, local parameters, parameter and species assignment rules, parameter and species initial assignments, species rate rules and function definitions. Models containing SBML events or parameter rate rules are not covered. Neither are models with irrational parameter values.

## 4 Conclusions

ODEbase as a canonical source of symbolic computation input related to existing models of biological processes offers a number of advantages:

1. *Interdisciplinary competence*: The derivation of adequate ODEs for the kinetics requires combined biological and mathematical expertise. Users can outsource this task to ODEbase.
2. *Economic use of human resources*: Symbolic computation input has been pre-computed and is directly available.
3. *Availability*: ODEbase models used and cited in the literature can be conveniently reviewed on the basis of the original data and re-used in follow-up publications.
4. *Canonical reference*: ODEbase fixes choices for the inevitable renaming of species and parameters to common mathematical notation. This facilitates comparability of results.
5. *Benchmarking*: ODEbase is perfectly suited to generate benchmark sets for novel algorithms and software in the field.
